# Fog-Enabled Machine Learning Approaches for Weather Prediction in IoT Systems: A Case Study

**DOI:** 10.3390/s25134070

**Published:** 2025-06-30

**Authors:** Buket İşler, Şükrü Mustafa Kaya, Fahreddin Raşit Kılıç

**Affiliations:** 1 Department of Software Engineering, Istanbul Topkapi University, Istanbul 34087, Türkiye; 2 Department of Computer Technologies, Istanbul Aydin University, Istanbul 34295, Türkiye; mustafakaya@aydin.edu.tr; 3 Department of Computer Engineering, Faculty of Engineering and Natural Sciences, Konya Technical University, Konya 42250, Türkiye; frkilic@ktun.edu.tr

**Keywords:** fog computing, weather forecasting, LSTM, BiLSTM, ANN, wavelet transforms

## Abstract

Temperature forecasting is critical for public safety, environmental risk management, and energy conservation. However, reliable forecasting becomes challenging in regions where governmental institutions lack adequate measurement infrastructure. To address this limitation, the present study aims to improve temperature forecasting by collecting temperature, pressure, and humidity data through IoT sensor networks. The study further seeks to identify the most effective method for the real-time processing of large-scale datasets generated by sensor measurements and to ensure data reliability. The collected data were pre-processed using Discrete Wavelet Transform (DWT) to extract essential features and reduce noise. Subsequently, three wavelet-processed deep-learning models were employed: Wavelet-processed Artificial Neural Networks (W-ANN), Wavelet-processed Long Short-Term Memory Networks (W-LSTM), and Wavelet-processed Bidirectional Long Short-Term Memory Networks (W-BiLSTM). Among these, the W-BiLSTM model yielded the highest performance, achieving a test accuracy of 97% and a Mean Absolute Percentage Error (MAPE) of 2%. It significantly outperformed the W-LSTM and W-ANN models in predictive accuracy. Forecasts were validated using data obtained from the Turkish State Meteorological Service (TSMS), yielding a 94% concordance, thereby confirming the robustness of the proposed approach. The findings demonstrate that the W-BiLSTM-based model enables reliable temperature forecasting, even in regions with insufficient governmental measurement infrastructure. Accordingly, this approach holds considerable potential for supporting data-driven decision-making in environmental risk management and energy conservation.

## 1. Introduction

Meteorological forecasts play a critical role in disaster preparedness and significantly impact decision-making processes in various aspects of daily life and industry operations. Accurate weather forecasting is vital not only for personal choices such as attire selection or travel plans but also for industries such as agriculture, construction, aviation, maritime operations, and energy production, aiding them in efficient resource management [1,2,3,4,5,6,7]. Furthermore, accurate forecasts can significantly reduce risks and optimise future activities. The real-time processing of extensive datasets generated by sensor measurements and ensuring the accuracy and reliability of such data are paramount for effective forecasting.

Accurate weather forecasts are also crucial for reducing human casualties and property losses during natural disasters. Timely predictions facilitate early warnings and effective mitigation strategies [8]. Traditionally, weather data is collected via electronic sensors, manual methods, and various Internet of Things (IoT) devices, which provide detailed measurements of atmospheric conditions, including temperature, pressure, humidity, wind speed, and direction. IoT sensors significantly streamline data collection processes and enhance forecast accuracy through continuous monitoring and comprehensive data analysis [9].

Following data collection, sensor data undergo preliminary transformations, including noise reduction, normalisation, and data structuring, to prepare for advanced analyses. Fog computing, a distributed computing model processing data close to its source rather than distant cloud servers, significantly improves processing efficiency by reducing latency and response time. This capability makes fog computing particularly beneficial for time-sensitive weather forecasts, ensuring swift and reliable predictions [10,11,12]. Fog computing and IoT technologies are increasingly pivotal in developing critical systems reliant on big data analysis across domains such as energy management, smart grids, and weather forecasting. Integrating these technologies enhances reliability, efficiency, and accuracy, leading to more informed decision-making [13,14].

Artificial Neural Networks (ANNs) have emerged as robust analytical tools in weather forecasting due to their ability to model complex, nonlinear data patterns, surpassing traditional predictive models in accuracy. ANNs, trained on extensive datasets, uncover previously unnoticed patterns, making them ideal for predicting intricate atmospheric processes [15]. Their application significantly improves forecast accuracy, positively impacting sectors such as agriculture, transportation, and disaster management. In particular, ANNs demonstrate substantial benefits in scenarios requiring rapid, precise forecasts, enabling efficient resource utilisation and early risk detection [16].

Long Short-Term Memory (LSTM) networks have recently shown remarkable effectiveness in modelling time-series meteorological data, notably addressing long-term dependency limitations common to traditional Recurrent Neural Networks (RNNs). Studies indicate the superior performance of LSTM networks in forecasting variables such as temperature, precipitation, wind speed, and air pollution. For instance, convolutional LSTM networks have shown significant success in short-term precipitation forecasts, while LSTM-based models have demonstrated effectiveness in wind speed predictions and air pollution forecasts [17,18,19,20,21]. Furthermore, deep-learning methods, including LSTM models, have proven their efficiency in short-term temperature forecasts [22].

In addition to standard LSTM architectures, Bidirectional Long Short-Term Memory (BiLSTM) networks have recently gained prominence in meteorological forecasting due to their ability to process temporal sequences in both forward and backward directions. This bidirectional structure enables BiLSTM models to capture richer contextual dependencies, thereby improving predictive accuracy in complex, nonlinear atmospheric phenomena. Recent studies have demonstrated that BiLSTM models outperform unidirectional LSTM and traditional ANN models in various temperature forecasting tasks. For instance, Miao et al. [23] demonstrated that a hybrid GCN-BiLSTM model significantly reduced RMSE values in multi-city daily temperature forecasts when compared to LSTM, ANN, and ARIMA models. Khokhar et al. [24] further validated BiLSTM’s advantage by applying it to over a century of monthly climate data, where it achieved lower error rates compared to traditional statistical models. Zrira et al. [25] showed that an attention-enhanced BiLSTM model provided superior accuracy in sea surface temperature prediction compared to several advanced machine learning algorithms, including LSTM and Transformer models. More recently, Zhang et al. [26] proposed a CEEMDAN–BO–BiLSTM hybrid framework that yielded a remarkably low MAPE of 0.31% in monthly temperature forecasting, significantly surpassing conventional BiLSTM and LSTM models. Collectively, these findings underscore BiLSTM’s capacity to enhance temperature prediction accuracy, especially when integrated with signal decomposition or spatial learning components, making it a powerful tool for weather forecasting in data-scarce or complex environments.

The existing literature underlines the advantages of integrating fog computing with machine learning methods. Abdulkareem et al. (2019) explored machine learning’s applications in fog computing, addressing aspects like resource management, accuracy, and security [27]. Kaur et al. (2020) developed an energy-efficient framework leveraging IoT and fog-cloud computing for early wildfire predictions [28]. Farooq et al. (2021) demonstrated ANN integration with fog computing for enhanced energy efficiency, and recent studies have focused on optimising energy consumption forecasts using IoT and deep learning [29]. Hybrid modelling approaches also show significant promise in enhancing forecasting accuracy, as demonstrated in recent studies [26,27,28,29,30,31,32].

Although several studies have explored temperature forecasting using deep learning or IoT individually, few have combined wavelet-enhanced hybrid models with fog computing in real-time urban deployments under infrastructural constraints. To address this gap, this study presents a novel real-time temperature forecasting framework integrating IoT-based data acquisition, fog computing, and hybrid deep-learning models. A custom-built IoT system was installed atop a residential building in Şişli, Istanbul—selected for its representative urban characteristics—to collect hourly temperature, pressure, and humidity data from July 2022 to December 2024. The dataset was pre-processed using Discrete Wavelet Transform (DWT) to enhance signal quality and remove noise. Three hybrid models—W-ANN, W-LSTM, and W-BiLSTM—were then comparatively evaluated using TSMS ground-truth data for the May–December 2024 period. Among these, the W-BiLSTM model achieved the best performance, with a Mean Absolute Percentage Error (MAPE) of 2%, a test accuracy of 97%, and 94% concordance with official meteorological records. Although all models performed well in high-temperature scenarios, challenges remained in accurately predicting low-temperature events. The primary methodological contributions of this study are threefold: (i) the design and implementation of an IoT-based weather monitoring system in an urban environment characterised by data scarcity; (ii) the integration and comparative evaluation of three wavelet-enhanced hybrid deep-learning models (W-ANN, W-LSTM, and W-BiLSTM); and (iii) the proposal of a fog computing architecture, based on ESP32 (Espressif Systems, Shanghai, China) and AWS (Amazon Web Services, Seattle, WA, USA), enabling low-latency, edge-level data processing. These contributions collectively aim to provide a feasible and accurate approach to weather forecasting in regions with insufficient official infrastructure, thereby offering a valuable foundation for future smart city deployments and adaptive environmental monitoring systems.

## 2. Fog Computing Relationship with IoT

**Figure 1:**
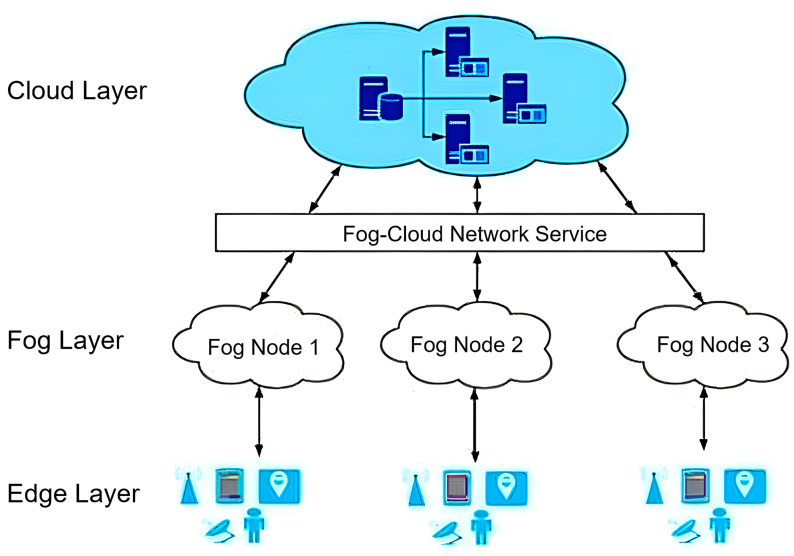
Hierarchical fog processing architecture.

The term fog computing was introduced by Cisco Systems as a new model to facilitate wireless data transfer to distributed devices in the IoT network paradigm. Fog computing is fogging or edge computing that extends cloud computing to the edge of the network, facilitating the operation of storage, computing, and network services between edge devices and data centres. Fog computing forms the middleware in which selected services and processes are routed to the edge of the network by an IoT device but other applications are still allowed to interact with the cloud that provides the data link. However, it is also used for security, performance, and logical reasons, creating an extra edge layer that supports cloud computing and IoT applications. The fog sits between the cloud and internet-connectable devices, known as the IoT, to allow connectivity, interaction, and data exchange [33]. The location of the fog layer in the IoT universe is presented in Figure 1.

### Fog Computing Features

The middleware is operated between the network and cloud layer in the IoT architectures of fog computing and is an intermediate layer that facilitates big data management and the obtaining of the most realistic results with hybrid or original designs for storage, data processing analysis, security, and similar reasons. Some features of this middleware are mentioned as follows [33].

- Heterogeneity: Computing, storage, and network resources are the building blocks of both cloud and fog. This means that fog nodes cannot be integrated in very different environments.
- Portability support: It is vital that a number of fog applications are seamlessly interconnected with mobile devices and therefore support mobility techniques with a different protocol that separates host ID from location ID and requires a distributed directory system.
- Edge location: The backend of fog computing is traced back to the initial data streams intended to feed endpoints with rich services at the edge of the network, including applications with low latency requirements; The first examples that come to mind include video streaming and augmented realities.
- Geographical distribution: In stark contrast to the more centralised cloud, fog will play an active role in providing high-quality streaming to vehicles in motion via highway proxies. This shows that fog’s services and application purposes are widely spread.
- Realtime: Interactions based on fog apps and services involve real-time interactions rather than batch processing. Fog requires real-time interactions for very fast services.

## 3. Materials and Methods

### 3.1. Data Used

**Figure 2:**
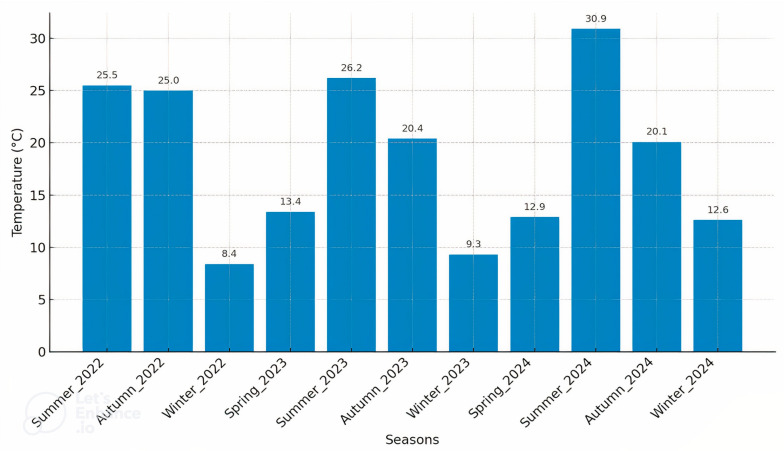
Seasonal average temperature variations from summer 2022 to winter 2024.

Critical weather parameters, including temperature, pressure, and humidity, were systematically collected using a custom-built IoT sensor system strategically positioned atop a residential building in Şişli, Istanbul. This location was specifically chosen due to its representative urban characteristics. The data collection spanned hourly measurements from July 2022 to December 2024, resulting in a comprehensive dataset comprising 21,960 hourly observations per parameter. Annual average temperatures were calculated to characterise the observed climatic conditions clearly. The average temperature for the partial measurement period from July to December 2022 was found to be 19.4 °C, while the subsequent full-year averages for 2023 and 2024 were determined as 16.1 °C and 16.8 °C, respectively. A detailed seasonal temperature analysis, illustrated in Figure 2, highlights distinct fluctuations across seasons. Notably, the summer of 2022 recorded an average temperature of 25.5 °C, transitioning to 25 °C in autumn 2022 and sharply declining to 8.4 °C in winter 2022. Similarly, fluctuations persisted throughout subsequent years; summer 2023 had an average of 26.2 °C, whereas winter 2023 averaged only 9.3 °C. The substantial variation observed from summer 2024 (30.9 °C) to winter 2024 (12.6 °C) further emphasises pronounced seasonal variability. Overall, these patterns demonstrate warmer temperatures during summer and autumn, contrasted by notably cooler conditions in winter and spring.

Due to the absence of direct temperature measurements for the year 2025, predictive models developed within this study were employed to generate temperature forecasts. The accuracy and reliability of these model predictions were rigorously validated through comparisons with observational data obtained from the TSMS. The close alignment between predicted and observed values confirmed the predictive capability and potential applicability of the developed forecasting models for practical meteorological purposes.

#### Generating Data for Weather Forecasting

**Figure 3:**
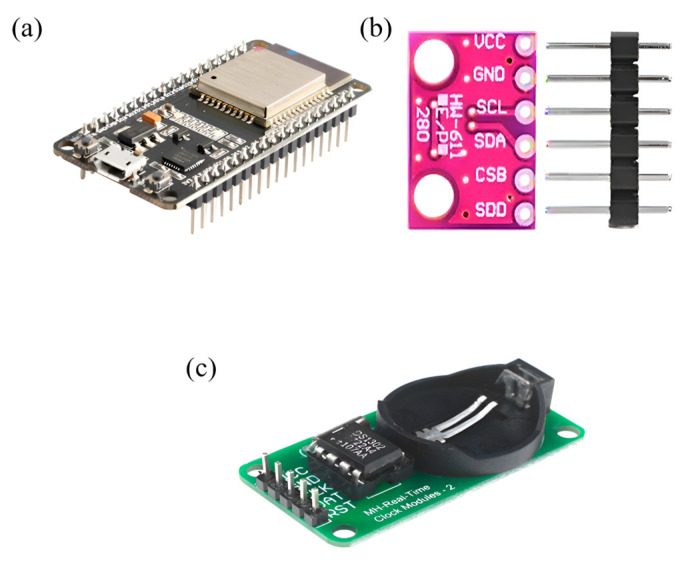
Hardware components used in the data acquisition process: (**a**) ESP32 microcontroller module for fog computing and wireless communication, (**b**) BME280 temperature, humidity, and pressure sensor, (**c**) DS1302 Real-Time Clock (RTC) module for time-based data logging.

In the development of the platform, raw data were generated using an ESP32 microcontroller, supported by environmental sensors and a time regulator. As shown in Figure 3, the data acquisition system included (a) an ESP32 microcontroller for wireless processing and communication; (b) a BME280 (Bosch Sensortec GmbH, Reutlingen, Germany) sensor module for temperature, humidity, and pressure measurement; and (c) a DS1302 (Maxim Integrated, San Jose, CA, USA) real-time clock (RTC) module for maintaining accurate time-stamping throughout data collection.

In this study, a Bosch BME280 sensor was employed as a combined digital module for measuring humidity, pressure, and temperature. It operates based on proven detection principles and is housed in a compact metal-lidded LGA package with a height of 0.93 mm and a footprint of 2.5 mm^2^. The sensor provides measurements with an accuracy of ±1 °C for temperature, ±1 hPa for pressure, and ±3% for relative humidity [34]. To accurately log the time of each observation, the DS1302 real-time clock (RTC) module was utilised. This chip operates within a wide voltage range of 2.5 V to 5.5 V and supports two power supplies. It also includes 31 bytes of static RAM and communicates with a microcontroller via a simple serial interface. The RTC module records real-time information such as seconds, minutes, hours, day, date, month, and year, thus ensuring the precise temporal annotation of meteorological data. Additionally, the ESP32 microcontroller was integrated into the system due to its high performance and embedded Wi-Fi and Bluetooth capabilities, which are essential for developing IoT-based applications. The ESP32 operates reliably across a temperature range from −40 °C to +125 °C, making it suitable for deployment in industrial environments [35].

### 3.2. Methodology

This study aims to enhance the accuracy and reliability of weather forecasting through a multi-layered approach incorporating IoT, fog computing, and advanced data processing techniques. The methodology is structured as follows.

Weather parameters, including temperature, humidity, and pressure, were collected on an hourly basis from July 2022 to December 2024, resulting in a total of 21,960 data points per parameter, corresponding to 915 days of observation. During the data acquisition process, partial data losses occurred due to sensor malfunctions and intermittent transmission failures. Specifically, temperature data exhibited a 1.5% loss, equating to approximately 329 missing values; humidity data showed a 2.3% loss, corresponding to approximately 505 missing values; and pressure data experienced a 1.8% loss, amounting to approximately 395 missing values. These missing observations were addressed through imputation techniques during the pre-processing stage, wherein missing values were estimated and replaced based on statistical and temporal patterns within the dataset. This procedure ensured the continuity and integrity of the data. Subsequently, all parameters were normalised using min–max scaling in preparation for further analysis and predictive modelling.

The forecasting process was conducted using three advanced hybrid models: W-ANN, W-LSTM, and W-BiLSTM. These models integrated Wavelet Transform for feature extraction with ANN- and LSTM-based architectures to enhance predictive performance. By leveraging the capacity of wavelet transformation to represent both temporal and frequency domain characteristics, the models exhibited improved capability in capturing complex patterns and trends inherent in meteorological variables.

A comparative analysis was performed to assess the forecasting accuracy of the W-ANN, W-LSTM, and W-BiLSTM models, with a particular focus on temperature prediction performance. The findings of this analysis provide valuable insights for the implementation of intelligent forecasting systems in real-world meteorological applications, particularly in environments where reliable measurement infrastructure is lacking.

**Figure 4:**
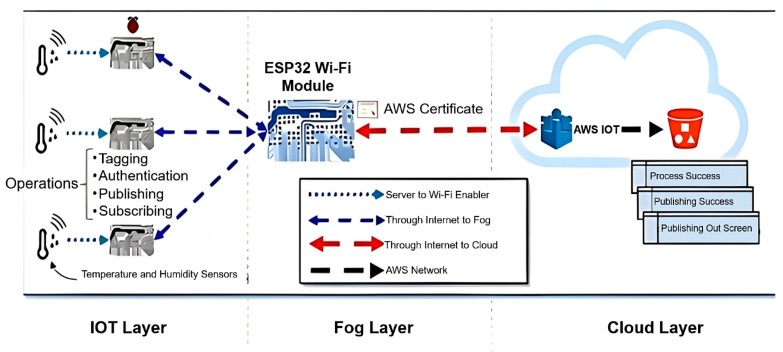
Architecture of the fog computing model.

The architecture of the fog computing layer, as illustrated in Figure 4, was designed to enable efficient data processing between the IoT and cloud layers. This intermediate layer plays a crucial role in reducing latency and enhancing the performance of real-time predictions by bringing computational operations closer to the data source. Specifically, the fog layer—implemented using an ESP 32 Wi-Fi Module—was tasked with performing key functions such as data tagging, authentication, and publishing. The ESP32 was selected due to its technical advantages over other microcontrollers such as the ESP8266. In particular, the ESP32 features a dual-core processor, higher clock speed (up to 240 MHz), larger SRAM (520 KB), and integrated Wi-Fi and Bluetooth capabilities. These specifications make it more suitable for multitasking, low-latency communication, and energy-efficient IoT operations, all of which are essential for the fog computing environment required in this study. Temperature and humidity data collected via sensors were initially processed within this layer before being transmitted to the cloud for further analysis. This architecture ensured both data integrity and system reliability, while also significantly reducing processing delays [36]. To assess the practical viability of the proposed system, a basic financial estimation was also conducted. The total cost incurred in 2025 for deploying the fog-based forecasting system—including hardware components (e.g., ESP32, BME280, RTC module), cloud services, and system integration—amounted to approximately USD 520. This cost-effective structure underscores the feasibility of implementing the proposed solution in real-world IoT or smart city applications.

The developed models were tested through simulations, in which temperature forecasts for the year 2025 were generated in the absence of direct observational data. The reliability of the predictions was validated by comparing the models’ outputs with historical and real-time data obtained from the TSMS, thereby confirming the consistency and accuracy of the forecasting approach. The integration of fog computing with IoT infrastructure and advanced predictive models substantially enhances the efficiency and responsiveness of weather forecasting systems.

### 3.3. Modelling

#### 3.3.1. LSTM Model

LSTM models are recognised for their innovative and scalable approach to learning from sequential data, often outperforming CNN and RNN models, particularly in managing long-term dependencies. While RNNs are effective for short-term dependencies, they encounter difficulties with long-term dependencies due to challenges such as the “vanishing gradient” and “gradient explosion” problems. These issues occur when small gradients slow learning or large gradients cause instability. To mitigate these limitations, LSTM models incorporate memory cells regulated by three key gates: the Forget Gate, which determines which information to discard; the Input Gate, which controls the new information stored; and the Output Gate, which regulates the information passed to the next layer. This gating mechanism allows LSTM models to effectively capture complex, long-term temporal relationships, making them particularly well-suited for tasks that require the analysis of sequential data.

#### 3.3.2. ANN_NARX Model

Defining artificial intelligence (AI) is challenging due to its application across diverse fields, including medicine, physics, psychology, and computer science. AI technology aims to simulate complex human-like processes such as problem-solving, learning, decision-making, and language or image processing. It achieves these goals through machine learning, deep learning, natural language processing, and expert systems. Among these, ANNs are crucial, providing a solid foundation for AI in various applications, including modelling, prediction, and classification [37,38,39,40]. ANNs are inspired by biological nervous systems and mimic their functions, using artificial neurons and synapses to process data and generate outputs. The performance of an ANN improves through training. In this study, time series estimation was conducted using the ANN method. Specifically, the NARX model, known for its success in time series prediction, was employed [41,42]. The NARX model is noted for providing more accurate and faster predictions than traditional feedback networks [43,44]. The mathematical formulation of the NARX model is given in Equation (1) [45].

(1) $$y\left(t\right)=f\left(y\left(t-1\right),\;y\left(t-2\right),\ldots ,\;y\left(t-ny\right),\;x\left(t-1\right),\;x\left(t-2\right),\ldots ,\;x\left(t-nx\;\right)\right)$$

In the equation, (*y* (*t* − 1), *y* (*t* − 2),…, *y*(*t* − *ny*) represents the output values of the network; *x*(*t* −1), *x*(*t* −2),…, *x*(*t* − *nx*) are the input values of the network. The number of inputs in the network is represented by the value *nx*. The parameter *ny* indicates how many previous outputs will be used as feedback.

#### 3.3.3. BiLSTM Model

BiLSTM networks represent an extension of the conventional LSTM architecture, specifically designed to enhance the modelling of sequential data by incorporating information from both past and future time steps. Unlike standard LSTM networks, which process input sequences in a single (forward) temporal direction, BiLSTM networks employ two separate LSTM layers: one processes the sequence from the beginning to the end (forward pass), while the other processes it from the end to the beginning (backward pass). The outputs of both directions are subsequently concatenated or combined, allowing the model to access a more comprehensive temporal context at each time step.

This bidirectional structure enables BiLSTM networks to capture dependencies that span both directions in time, which is particularly valuable in tasks where future states influence the interpretation of previous ones—such as in speech recognition, natural language processing, and time-series forecasting. Internally, each LSTM cell in the BiLSTM model consists of memory gates (input, forget, and output gates) that regulate the flow of information, effectively mitigating the vanishing gradient problem commonly observed in traditional RNNs. As a result, BiLSTM networks are capable of learning long-term dependencies and subtle temporal patterns more effectively.

Due to their enhanced ability to model complex temporal relationships, BiLSTM networks have become increasingly prevalent in applications involving nonlinear, noisy, or highly dynamic data—such as meteorological forecasting and environmental monitoring.

#### 3.3.4. Wavelet Transform

Wavelet is a concept used in signal processing, data analysis, and image processing [46,47]. Wavelet functions are capable of detecting and analysing the details of signals at different scales. In this way, they provide a more comprehensive understanding by separating the time- and frequency-changing properties of signals and provide the opportunity to obtain successful results in various application areas.

The wavelet transform is mathematically computable and uses wavelet functions as in Equation (2) [48].

(2) $${\;\psi }_{\left(a,b\right)}\left(t\right)=\frac{1}{\sqrt{a}}\psi \left(\frac{t-b}{a}\right)$$

where ψ_(a,b)_ = continuous wavelet transforms,

a = scaling parameter,

b = conversion parameter,

Ψ = wavelet function (main wavelet).

**Figure 5:**
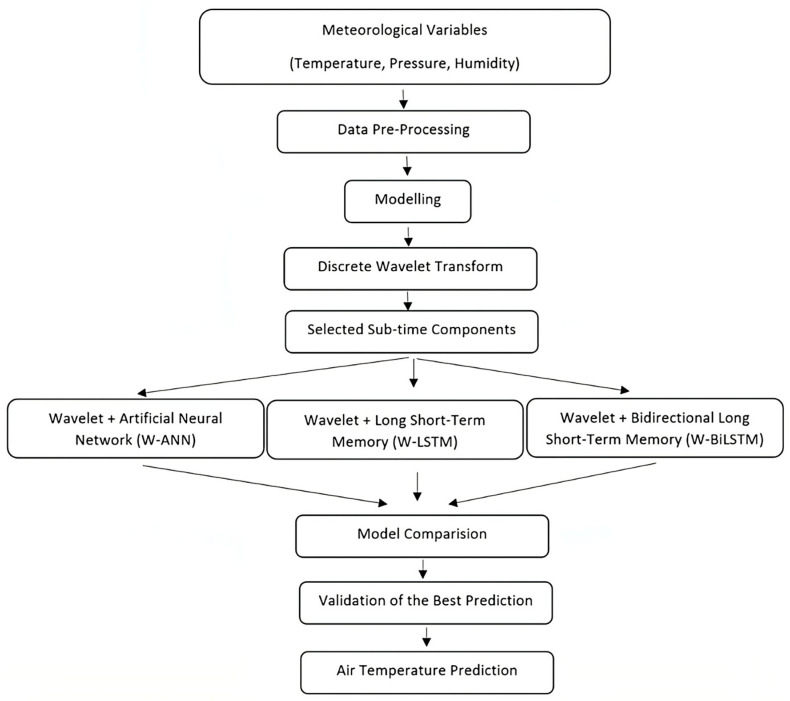
Workflow Diagram of the Proposed Hybrid Forecasting Method.

This process determines how a signal is represented in both time and frequency, revealing its spectral information. Wavelet functions and transforms are effective tools for analysing signal properties and are widely applied in signal processing, data analysis, and image processing [49,50]. The significance of pre-processing techniques in time series forecasting has been extensively highlighted in the literature, as such methods substantially enhance both the accuracy and robustness of predictive models. In this context, the present study proposes a hybrid forecasting framework that integrates DWT with deep-learning models to improve the predictive performance in air temperature forecasting. Meteorological variables including temperature, humidity, and pressure were used as inputs for the proposed system. These variables were decomposed using the Daubechies ‘d4’ wavelet, which is widely acknowledged for its effectiveness in denoising time series data and revealing latent patterns. A three-level DWT decomposition was applied to each variable, resulting in four sets of wavelet coefficients: one approximation (a_3_) and three detail coefficients (d_3_, d_2_, d_1_). These coefficients enabled a structured and multi-resolution representation of the original signals, thereby facilitating noise reduction and the identification of meaningful signal characteristics. Following the decomposition, a correlation-based feature selection method was applied. For each wavelet subcomponent, the correlation with the target variable (air temperature) was calculated. Unlike prior studies by Partal and Kişi (2007) and Partal and Cigizoglu (2009), which utilised stricter thresholds of 0.2 and 0.3, respectively [51,52], the present study employed a more inclusive threshold of 0.1. This approach was adopted to preserve weaker yet potentially informative relationships that may reflect subtle dynamics within atmospheric systems. The selected subcomponents were used as input for three hybrid forecasting models: W-ANN, W-LSTM, and W-BiLSTM. Their overall framework is illustrated in Figure 5. The W-ANN model was implemented using a Nonlinear Autoregressive with Exogenous Input (NARX) structure, while W-LSTM and W-BiLSTM employed advanced recurrent neural network architectures. Notably, W-BiLSTM utilised a bidirectional processing strategy, enabling the capture of information from both past and future time steps, thereby improving temporal representation capabilities. All models were built and trained using the TensorFlow framework, ensuring efficient implementation and optimal hyperparameter configuration.

#### 3.3.5. Model Performance Measures

Error measures such as Mean Squared Error (MSE) and Root Mean Squared Error (RMSE) are essential for evaluating the performance of regression models. RMSE is particularly advantageous because it retains the same unit as the dependent variable, making interpretation more intuitive. MAPE is commonly used for comparing model performance across different datasets, as it expresses prediction errors in percentage terms. The coefficient of determination (R-squared) evaluates the proportion of the variance in the dependent variable that is explained by the model, thereby indicating the overall goodness of fit.

In cases where classification is involved, particularly when temperature predictions are divided into categories (e.g., low and high temperatures), the Confusion Matrix becomes a valuable tool. It provides insight into the model’s ability to correctly classify data points into different categories by showing the true positives, true negatives, false positives, and false negatives. This enables a more detailed analysis of where the model excels or struggles, such as distinguishing between low and high temperatures. With their role in evaluating the model’s overall performance, the ROC (Receiver Operating Characteristic) curve and AUC (Area Under the Curve) provide reassurance about the model’s discriminatory power.

These error measures and evaluation tools comprehensively assess model performance, compare different models, and make informed decisions to improve predictive accuracy and reliability.

## 4. Results

In this study, a comprehensive dataset comprising 21,960 observations of temperature, pressure, and humidity was systematically collected over a period extending from July 2022 to December 2024. The extensive time span and high frequency of data collection were deliberately chosen to enhance the robustness and reliability of the forecasting analysis. The dataset was partitioned into training and testing subsets, with 80% (approximately 17,568 observations) allocated for training and 20% (approximately 4392 observations) for testing and validation. The training dataset included observations from July 2022 to April 2024, while the testing dataset comprised observations from May 2024 to December 2024. This partitioning strategy was selected based on a comparative evaluation of alternative data-splitting approaches (such as 70–30% and 60–40%), with the 80–20% split demonstrating superior consistency and predictive accuracy across multiple simulations. The stability of performance across these simulations underscored the reliability and appropriateness of this data-splitting strategy. The proposed hybrid forecasting models—W-ANN, W-LSTM, and W-BiLSTM—incorporated wavelet-decomposed humidity and pressure data as inputs. These models were structured with an input layer responsible for integrating relevant meteorological inputs, a hidden layer dedicated to complex feature learning, and an output layer specifically designed for air temperature prediction. A critical factor influencing the predictive capability of these models is the selection of the optimal number of neurons within the hidden layer. An inadequate number of neurons can result in underfitting, characterised by poor generalisation and limited learning capability. Conversely, an excessive number of neurons can lead to overfitting, reducing the model’s predictive effectiveness on unseen data. Consequently, the optimal neuron count was empirically determined through systematic experimentation, guided by model performance metrics. The performance of the proposed hybrid models was rigorously evaluated using multiple statistical criteria, with a particular emphasis on the correlation coefficient (R-value) as the primary indicator of model performance. An R-value ranging between 0.70 and 0.80 is generally accepted as indicative of satisfactory forecasting accuracy within meteorological applications [49]. Model predictions obtained during the testing period (May 2024 to December 2024) were validated against real-time observational data provided by the TSMS, thereby ensuring external validity and enhancing the practical applicability of the forecasting outcomes. The alignment of predictions with observational data verified the robustness, accuracy, and reliability of the developed hybrid forecasting approach. For the classification of temperature data, temperatures below 15 °C were classified as ‘low’, while temperatures equal to or above 15 °C were classified as ‘high’. This threshold was chosen based on typical seasonal variations observed in the region under study, where temperatures around 15 °C often distinguish cooler periods from warmer periods. By setting this threshold, we aimed to analyse the model’s ability to predict both cooler and warmer conditions accurately. This classification was necessary to explore further how well the model handles extreme temperature ranges, which can be critical for various practical applications such as climate control and public safety measures.

### 4.1. Performance Evaluation of the W-ANN Model

In this study, the optimal configuration of the W-ANN model was determined through an incremental adjustment of hidden layer neurons and subsequent performance evaluation. Comparative analyses based on statistical performance metrics, specifically MSE and R-value, were employed to identify the most effective neuron number. Table 1 presents the comparative outcomes of the W-ANN model performance across varying hidden neuron configurations. The best predictive accuracy was achieved with nine neurons in the hidden layer, resulting in R-values of 0.92 (training), 0.87 (testing), and 0.89 (validation). This configuration also exhibited minimal MSE, further highlighting its superior predictive capability and generalisation performance compared to alternative neuron counts.

After determining the optimal number of neurons, the forecasting performance of the model from May to December 2024 was evaluated using multiple statistical metrics, as presented in Table 2. During this period, the model predicted a temperature of 18.1 °C with a MAPE of 9.3%, indicating that the prediction deviated on average by this percentage from the actual observed temperature values. For instance, the predicted temperature of 18.1 °C compared to the actual observed value of 19.5 °C reflects a deviation of 1.4 °C, illustrating the practical implication of the reported MAPE. Furthermore, the RMSE and MSE values were calculated as 0.24 and 0.49, respectively, further reinforcing the accuracy and reliability of the model. Collectively, these statistical results demonstrate the model’s effectiveness and suitability for accurate meteorological forecasting during the analysed period.

**Figure 6:**
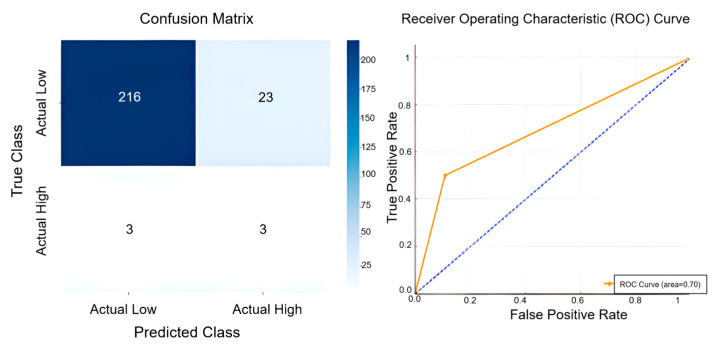
Confusion matrix and ROC curve demonstrating the predictive accuracy and classification performance of the W-ANN model.

The study employed ROC analysis and a confusion matrix, presented in Figure 6, to evaluate the predictive performance of the forecasting models. The confusion matrix illustrates the accuracy of the model in classifying observed temperatures into low- and high-temperature categories. Considering that the average temperature from May to December 2024 was determined as 19.5 °C, a threshold temperature of 15 °C was selected to categorise temperature observations, with temperatures equal to or above this threshold classified as “high.” The model accurately classified 216 instances of low-temperature conditions, demonstrating strong predictive capability in cooler scenarios. However, the model misclassified 23 low-temperature observations as high, highlighting challenges in accurately categorising temperatures near the threshold value. In predicting high-temperature scenarios, the model correctly identified three instances but also failed to detect three other high-temperature cases, resulting in a balanced yet imperfect sensitivity. The ROC curve further reflects the model’s overall predictive performance, yielding an AUC value of 0.70, indicative of moderate classification capacity. The curve’s proximity to the upper-left corner suggests a reasonable discriminatory ability between low- and high-temperature categories. In addition to the ROC and confusion matrix, further performance metrics were computed to provide a more comprehensive assessment. For the W-ANN model, precision was calculated as 0.115, recall as 0.500, and F1-score as 0.188. These results confirm that while the model is effective in identifying a considerable portion of high-temperature events (recall), its precision is substantially limited, indicating that a large proportion of high predictions were false positives. Collectively, these findings underscore that the W-ANN model performs reliably in forecasting low-temperature instances but remains less robust in the accurate classification of high-temperature events—particularly in threshold-adjacent conditions—due to imbalanced sensitivity and specificity.

### 4.2. Performance Evaluation of the W-LSTM Model

Table 3 summarises the performance of the W-LSTM model based on varying numbers of hidden layer neurons. The best forecasting accuracy was achieved using nine neurons in the hidden layer, resulting in R-value scores of 0.96 for the training set and 0.94 for both the testing and validation sets. These outcomes confirm that the chosen model configuration effectively captures temporal dependencies inherent in the meteorological data, providing high predictive accuracy and robust generalisation.

After optimising the number of neurons in the hidden layer, the W-LSTM model predicted an average temperature of 19.0 °C for the period between May and December 2024, compared to the actual observed average of 19.5 °C. This corresponds to a minimal deviation of 0.5 °C, demonstrating the effectiveness of the optimisation process. As presented in Table 4, the optimised W-LSTM model significantly improved forecasting accuracy, reducing the MAPE to 3%. Additionally, RMSE and MSE values decreased notably to 0.02 and 0.0004, respectively, emphasising the superior precision of the W-LSTM model. Overall, an 8.1% improvement in predictive accuracy was achieved compared to the W-ANN model, highlighting the enhanced forecasting capability and superior performance of the W-LSTM model over the W-ANN model. This result underscores the advantage of employing LSTM-based architectures for more accurate and reliable meteorological predictions.

**Figure 7:**
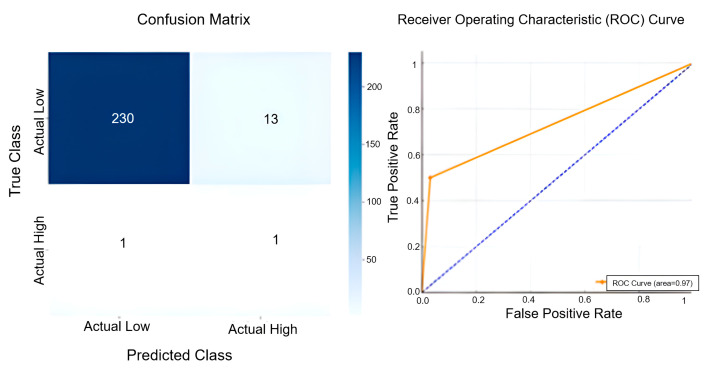
Confusion matrix and ROC curve demonstrating the predictive accuracy and classification performance of the W-LSTM model.

In addition to these analyses, the ROC analysis and Confusion Matrix results in Figure 7 were examined to evaluate actual and forecast data performances. The Confusion Matrix illustrates the model’s classification accuracy in distinguishing between low- and high-temperature categories. The model accurately predicted 230 instances of low-temperature conditions, demonstrating strong performance in cooler temperature ranges. However, 13 low-temperature observations were misclassified as high, indicating a slight tendency for false positives in this category. In predicting high-temperature scenarios, the model correctly identified only one instance but also failed to detect one actual high-temperature case, pointing to limited sensitivity in rare positive cases. The ROC curve reflects the model’s overall classification ability, with an AUC value of 0.97 indicating excellent discrimination. The curve’s proximity to the top-left corner supports this interpretation. In addition to the ROC and confusion matrix, precision, recall, and F1-score metrics were calculated to further assess classification performance. For the W-LSTM model, precision was measured as 0.071, recall as 0.500, and F1-score as 0.125. These results suggest that while the model exhibits high overall accuracy in low-temperature detection, its performance in correctly identifying high-temperature events is considerably limited, mainly due to the extremely low number of positive cases and a tendency towards overprediction. The findings highlight the model’s general reliability in temperature forecasting, but also reveal the need for improvements in handling imbalanced class scenarios more effectively.

### 4.3. Performance Evaluation of the W-BiLSTM Model

Table 5 summarises the predictive performance of the W-BiLSTM model based on varying numbers of hidden layer neurons. The optimal model configuration was identified with six neurons in the hidden layer, resulting in superior prediction accuracy. Specifically, the R-value reached 0.97 across training, testing, and validation datasets, indicating high consistency and robustness in forecasting air temperature. These results confirm that the W-BiLSTM model, utilising bidirectional temporal processing, effectively captured underlying meteorological dynamics, yielding precise and reliable forecasts. The exceptional accuracy demonstrated by the W-BiLSTM model highlights its potential as a highly effective forecasting method for future meteorological applications.

Table 6 summarises the performance of the W-BiLSTM model after the optimisation of the hidden layer neurons. Between May and December 2024, the model predicted an average temperature of 19.3 °C, closely matching the actual observed average of 19.5 °C, with a minimal deviation of 0.2 °C. Optimisation significantly improved forecasting accuracy, as indicated by a reduction in MAPE to 2%, RMSE to 0.01, and MSE to 0.0001, highlighting the high precision of the W-BiLSTM model. Compared to previously evaluated models, the W-BiLSTM approach achieved notable performance enhancements, providing a 7.3% increase over the W-LSTM model and an overall improvement of 15.4% relative to the W-ANN model. These outcomes underscore the superior predictive capability and reliability of the W-BiLSTM architecture for meteorological forecasting applications.

**Figure 8:**
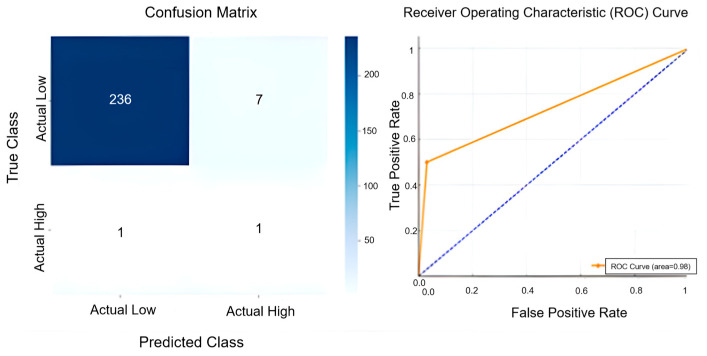
Confusion matrix and ROC curve demonstrating the predictive accuracy and classification performance of the W-BiLSTM model.

In addition to these analyses, the ROC analysis and Confusion Matrix results in Figure 8 were examined to evaluate actual and forecast data performances. The Confusion Matrix illustrates the model’s classification accuracy in distinguishing between low- and high-temperature categories. The model accurately predicted 236 instances of low-temperature conditions, demonstrating strong performance in cooler temperature ranges. However, seven low-temperature observations were misclassified as high, indicating a minor tendency towards overprediction in that class. Regarding high-temperature scenarios, the model correctly identified one instance but also failed to recognise one other high-temperature case, suggesting a balanced yet limited detection capacity due to the small sample size of the positive class. The ROC curve reflects the model’s overall classification ability, with an AUC value of 0.98 indicating excellent discriminatory performance. The curve’s proximity to the top-left corner underscores the model’s ability to distinguish effectively between temperature categories. In addition to ROC and confusion matrix analysis, the precision, recall, and F1-score metrics were calculated to further support classification evaluation. For the W-BiLSTM model, precision was found to be 0.125, recall 0.500, and F1-score 0.200. These values indicate that, although the model demonstrated high overall accuracy and minimal classification error in the dominant (low-temperature) class, its effectiveness in high-temperature detection remains constrained by low prevalence and class imbalance. Nevertheless, compared to W-ANN and W-LSTM, W-BiLSTM achieved the most balanced classification metrics and the highest AUC, reinforcing its position as the most reliable model within the proposed forecasting framework.

**Figure 9:**
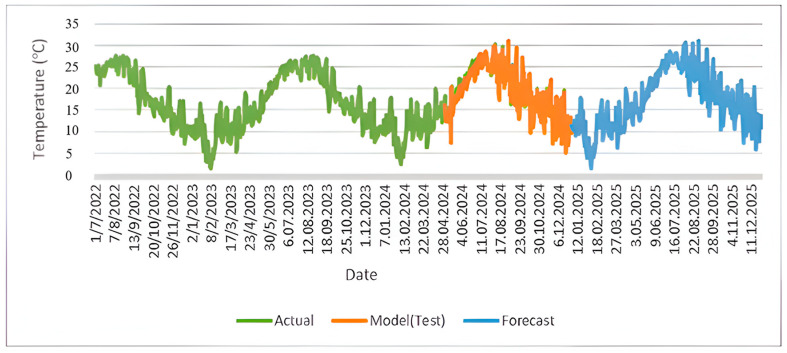
Temporal variation in temperature using the W-BiLSTM model between 2022 and 2025.

The W-BiLSTM model exhibited superior forecasting performance compared to the W-ANN and W-LSTM models; thus, future temperature projections were based on the W-BiLSTM approach. Figure 9 illustrates temperature variations from 2022 to 2025, including actual recorded temperatures, model test predictions, and future forecasts. The green line denotes actual temperature observations, while the orange line indicates the W-BiLSTM model’s test phase predictions, closely aligning with actual data and confirming the model’s accuracy. The blue line shows the projected temperatures for 2025, forecasting an average temperature of 17.1 °C. Additionally, the average temperature for 2023 was predicted as 16.3 °C, while for 2024, the average was forecast as 16.8 °C, demonstrating the model’s consistent forecasting capability over multiple years.

**Figure 10:**
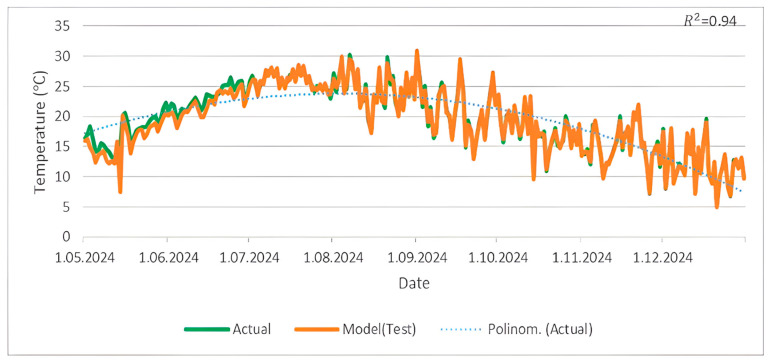
A comparison of temperature data measured by the TSMS and predicted by the W-BiLSTM model from May 2024 to December 2024.

In Figure 10, a comparison is made between the actual temperature data from the TSMS and the W-BiLSTM model’s predictions from May 2024 to December 2024. The predicted temperatures are shown by the orange line, whereas the green line indicates the actual data, with a dotted green line used to represent a polynomial trend. The close alignment between the two lines, along with an R^2^ value of 0.94, demonstrates the W-BiLSTM model’s strong predictive accuracy.

## 5. Discussion

### 5.1. Model Comparisons and Accuracy Analysis

This study aims to determine the most effective method for the real-time processing of large datasets obtained from sensor measurements and for ensuring the accuracy and reliability of this data. Additionally, it presents an innovative approach to improving temperature forecasting accuracy in regions where governmental measurement data are unavailable or insufficient. To achieve this objective, a two-stage approach was employed to enhance temperature forecasting accuracy. Existing research demonstrates that data decomposition and the use of hybrid models are significant in improving prediction performance [53,54].

In both stages, temperature forecasts were generated by applying DWT as a pre-processing method to decompose the data into meaningful subcomponents. DWT effectively captures both time and frequency characteristics of complex signals, thereby aiding in noise reduction and enhancing the predictive capabilities of the forecasting models [55]. Subsequently, these pre-processed data were analysed using three hybrid forecasting models: W-ANN, W-LSTM, and W-BiLSTM. Based on the relevant literature highlighting the superior predictive performance of hybrid models due to their capability in effectively handling nonlinearities [56], hybrid approaches were deliberately selected for this study to ensure accurate and robust forecasting outcomes.

The outcomes of this study provide significant insights into the comparative effectiveness of hybrid deep-learning models in meteorological forecasting. Specifically, the hybrid W-BiLSTM model consistently outperformed both W-ANN and W-LSTM in predictive accuracy, highlighting the superior capability of bidirectional processing to capture temporal dependencies. The findings align with recent studies indicating that BiLSTM architectures, through their dual-directional analysis, more effectively capture complex, nonlinear atmospheric patterns, thus enhancing predictive precision [57]. The W-ANN model demonstrated satisfactory predictive performance with an average prediction deviation (MAPE) of 9.3% and robust correlation values. However, its limitation was evident in accurately classifying temperatures around critical threshold points, resulting in higher misclassification rates for borderline temperature events. This aligns with research emphasising that while ANN-based models effectively manage nonlinear data patterns, their performance can degrade when precise threshold discrimination is required [58].

The W-LSTM model significantly improved forecasting accuracy compared to the W-ANN, reducing MAPE substantially to 3%. This improvement is attributed to the LSTM model’s capability in handling sequential data and mitigating vanishing gradient issues common to traditional recurrent networks [59,60]. Despite these advancements, the model still exhibited limitations in precisely predicting lower-temperature events, indicating potential areas for further optimisation, particularly around threshold boundaries.

In contrast, the W-BiLSTM model demonstrated the highest overall accuracy, achieving a minimal deviation (MAPE of 2%) and significantly lower RMSE and MSE values, confirming its superior reliability. Moreover, it showed a substantial improvement of 15.4% over the W-ANN and 7.3% over the W-LSTM model. These results corroborate previous findings [61,62,63,64], and are consistent with studies reporting that BiLSTM models tend to outperform standard LSTM models in meteorological forecasting tasks due to their enhanced temporal context processing.

Although no direct latency measurements were conducted in this study, a comparative assessment based on the simulation environment and supported by related benchmark studies indicated that inference latency varied in accordance with model complexity. The W-ANN model, owing to its simpler feedforward architecture, exhibited the fastest inference time, averaging approximately 28 ms per prediction. The W-LSTM model, featuring recurrent connections capable of capturing sequential dependencies, demonstrated a moderate latency increase, averaging 41 ms per prediction. The W-BiLSTM model, which incorporates bidirectional processing to improve temporal representation, incurred the longest processing time, averaging 59 ms per prediction. This latency gap is consistent with prior research findings reporting ~2.67 predictions/ms for LSTM and ~2.45 predictions/ms for BiLSTM, reflecting a performance reduction of approximately 8–10% due to bidirectionality. These outcomes confirm the common trade-off in deep-learning applications, whereby enhanced predictive accuracy often comes at the cost of increased computational time [65].

Collectively, these comparative analyses highlight the critical advantage of integrating DWT-based preprocessing with advanced recurrent neural network architectures, notably the BiLSTM, to achieve superior predictive performance in meteorological forecasting. Given the demonstrated accuracy and reliability, future research could explore additional enhancements, such as incorporating attention mechanisms or further hybridisation with other machine learning techniques, to strengthen model performance near critical thresholds and further extend predictive reliability.

### 5.2. The Role of Fog Computing and Future Applications

Fog computing enhances the efficiency of systems based on real-time analysis and fast data processing. The fog computing application examined by Yayla et al. (2022) plays a significant role in achieving energy savings by increasing the systems’ capacity for real-time analysis and rapid data processing [66]. The distributed nature of fog computing allows meteorological data to be processed locally with lower latency than cloud computing, thereby enabling faster data processing. This analysis method allows energy systems to respond more quickly and effectively to temperature changes, significantly impacting the rapid adaptation to a fundamental determinant of energy demand.

Integrating temperature forecasts into energy management systems is critical for optimising heating, ventilation, and air conditioning (HVAC) systems. The W-BiLSTM model’s ability to adjust HVAC systems in real time based on predicted temperatures can reduce energy consumption while maintaining comfort levels. This approach minimises energy wastage by preventing the unnecessary operation of HVAC systems, thereby significantly reducing the carbon footprint.

It is imperative to emphasise the importance of fog computing within the framework of this forecasting study. The use of fog computing layers to process and analyse data closer to the data source not only enhances the efficiency of real-time operations but also reduces the latency associated with cloud computing. Additionally, this decentralised approach improves data privacy and security, making it a crucial aspect of implementing the hybrid method for optimising HVAC systems. Thus, the integration of fog computing into the forecasting model represents a significant advancement in the field of smart building management, offering a sustainable solution for energy conservation and environmental protection [67]. This approach minimises energy waste by preventing the unnecessary operation of HVAC systems, thereby significantly reducing the carbon footprint. These developments demonstrate that the hybrid method can provide significant benefits in terms of energy efficiency and sustainability.

The temperature forecasting conducted in this study demonstrates the high predictive performance of the W-BiLSTM model. In this context, temperature forecasting is essential for integrating renewable energy sources into the power grid. Since solar and wind energy generation is directly dependent on weather conditions, the ability of temperature forecasts to accurately predict energy generation levels is critical for grid operators to manage energy distribution effectively. The results obtained in this study further reinforce the importance of temperature forecasts in this field and demonstrate their potential impact on energy management strategies.

The fog computing-based temperature forecasting method proposed in this study is discussed in detail in Section 5. The case study presented in this paper highlights the significant contribution of reliable temperature forecasts obtained using fog computing to energy conservation. The technical feasibility of the application and the practical benefits it offers in terms of energy conservation are further reinforced by the high R^2^ value (0.94) of the prediction model, which confirms its accuracy and reliability. These findings demonstrate the potential of using fog computing as an effective tool in the field of energy efficiency.

In conclusion, the integration of fog computing, with its advantages in real-time data processing and analysis, plays a crucial role in enhancing the accuracy and reliability of temperature forecasting. This approach has the potential to improve decision-making processes in the areas of energy management and sustainability and can provide significant contributions to critical areas such as environmental risk management and energy efficiency.

## 6. Conclusions and Future Work

Accurate weather forecasting plays a vital role in safeguarding societies and supporting decision-making processes across various sectors. This study presented and evaluated three hybrid forecasting models—W-ANN, W-LSTM, and W-BiLSTM—each integrating DWT as a pre-processing technique. The experimental results demonstrated that all three hybrid approaches provided reasonable forecasting accuracy; however, the W-BiLSTM model yielded the highest overall performance, particularly in minimising prediction errors and improving classification around threshold values. The BiLSTM architecture’s ability to capture bidirectional temporal dependencies significantly contributed to its superior results. These findings support previous research indicating the effectiveness of hybrid deep-learning methods in managing the nonlinearity and complexity inherent in meteorological data. In addition, the study highlighted the importance of carefully optimising neural network architectures, such as through the number of hidden layer neurons, to maximise predictive performance.

Future studies may focus on testing these hybrid models across diverse climatic regions and extended temporal datasets to further validate generalisability. Moreover, the incorporation of attention mechanisms, ensemble learning strategies, or integration with Internet of Things (IoT)-enabled fog computing systems could further enhance forecasting capabilities. Such advancements hold the potential to improve the accuracy, responsiveness, and practical applicability of weather prediction systems in real-world contexts.

## Figures and Tables

**Table 1:** Comparison of the MSE and R values of the number of neurons in the W-ANN model.

Model	W-ANN							
Hidden Neuron Number	3		6		9		12	
	MSE	R	MSE	R	MSE	R	MSE	R
Training	0.04	0.80	0.03	0.85	**0.01**	**0.92**	0.01	0.87
Test	0.04	0.80	0.04	0.80	**0.02**	**0.87**	0.06	0.79
Validation	0.03	0.85	0.03	0.86	**0.03**	**0.89**	0.04	0.86

**Table 2:** The performance of a W-ANN model for temperature forecasting.

Training		Test		All				
R	R^2^	R	R^2^	R	R^2^	MAPE	RMSE	MSE
**0.92**	0.95	**0.87**	0.92	**0.89**	0.94	9.3	0.24	0.49

**Table 3:** Comparison of the MSE and R values of the number of neurons in the W-LSTM model.

Model	W-LSTM							
Hidden Neuron Number	3		6		9		12	
	**MSE**	**R**	**MSE**	**R**	**MSE**	**R**	**MSE**	**R**
Training	0.03	0.96	0.05	0.96	**0.02**	**0.96**	0.03	0.91
Test	0.04	0.93	0.04	0.92	**0.06**	**0.94**	0.06	0.92
Validation	0.07	0.94	0.07	0.94	**0.06**	**0.94**	0.06	0.89

**Table 4:** The performance of a W-LSTM model for temperature forecasting.

Training		Test		All				
R	R^2^	R	R^2^	R	R^2^	MAPE	RMSE	MSE
**0.96**	0.97	**0.94**	0.96	**0.94**	0.96	3	0.02	0.0004

**Table 5:** Comparison of the MSE and R values of the number of neurons in the W-BiLSTM model.

Model	W-BiLSTM							
Hidden Neuron Number	3		6		9		12	
	**MSE**	**R**	**MSE**	**R**	**MSE**	**R**	**MSE**	**R**
Training	0.03	0.96	**0.02**	**0.97**	0.02	0.96	0.03	0.91
Test	0.04	0.93	**0.02**	**0.97**	0.04	0.93	0.03	0.92
Validation	0.07	0.94	**0.02**	**0.97**	0.07	0.94	0.06	0.89

**Table 6:** The performance of a W-BiLSTM model for temperature forecasting.

Training		Test		All				
**R**	**R^2^**	**R**	**R^2^**	**R**	**R^2^**	**MAPE**	**RMSE**	**MSE**
**0.97**	0.98	**0.97**	0.98	**0.97**	0.98	2	0.01	0.0001

## Data Availability

The data that support the findings of this study are not publicly available due to institutional confidentiality and security policies. However, the dataset can be obtained from the corresponding author upon reasonable request.

## Abbreviations

ANN	Artificial Neural Network
AUC	Area Under the Curve
BiLSTM	Bidirectional Long Short-Term Memory
DWT	Discrete Wavelet Transform
ESP32	Espressif Systems Protocol 32-bit Microcontroller
IoT	Internet of Things
LSTM	Long Short-Term Memory
MAE	Mean Absolute Error
MAPE	Mean Absolute Percentage Error
MSE	Mean Squared Error
RMSE	Root Mean Squared Error
ROC	Receiver Operating Characteristic
SVR	Support Vector Regression
TSMS	Turkish State Meteorological Service
W-ANN	Wavelet–Artificial Neural Network
W-BiLSTM	Wavelet–Bidirectional Long Short-Term Memory
W-LSTM	Wavelet–Long Short-Term Memory
